# Identification of a novel mtDNA lineage B3 in chicken (*Gallus gallus domesticus*)

**DOI:** 10.24272/j.issn.2095-8137.2017.039

**Published:** 2017-07-18

**Authors:** Xun-He Huang, Gui-Mei Li, Xing Chen, Ya-Jiang Wu, Wei-Na Li, Fu-Sheng Zhong, Wen-Zhi Wang, Zhao-Li Ding

**Affiliations:** ^1^School of Life Sciences, Jiaying University, Meizhou Guangdong 514015, China; ^2^Kunming Biological Diversity Regional Center of Large Apparatus and Equipments, Chinese Academy of Sciences, Kunming Yunnan 650223, China; ^3^Public Technical Service Center, Kunming Institute of Zoology, Chinese Academy of Science, Kunming Yunnan 650223, China; ^4^State Key Laboratory of Genetic Resources and Evolution, Kunming Institute of Zoology, Chinese Academy of Sciences, Kunming Yunnan 650223, China; ^5^State Key Laboratory for Conservation and Utilization of Bioresource in Yunnan, Yunnan University, Kunming Yunnan 650091, China; ^6^Forensic Science Service of Yunnan Endangered Species Scientific Commission, Kunming Yunnan 650223, China

## DEAR EDITOR,

In this study, we sequenced the complete mitochondrial DNA genome (mitogenome) of the Zhengyang Yellow chicken (*Gallus gallus domesticus*) by next-generation sequencing technology. Samples were taken from Zhumadian city, Henan Province, China. The complete mitogenome was 16 785 bp in size, and had a nucleotide composition of 30.3% (A), 23.7% (T), 32.5% (C), and 13.5% (G), with a high AT content of 54.0%. The assembled mitogenome exhibited typical mitochondrial DNA (mtDNA) structure, including a non-coding control region, two rRNA genes, 13 protein-coding genes, and 22 tRNA genes. Phylogenetic analysis indicated that this mitogenome defined a novel sub-haplogroup B3 within haplogroup B. These results should provide essential information for chicken domestication and insight into the evolution of genomes.

Zhengyang yellow chicken (*Gallus gallus domesticus*) is an indigenous breed from Zhengyang County of Zhumadian in Henan Province, China (China National Commission of Animal Genetic Resources, 2011), and is noted for its yellow-colored shank, beak, and feathers. This chicken possesses many valuable and stable genetic traits that could be used as a gene bank for cultivating and creating new breeds in China. Here, for the first time, we sequenced and characterized the complete mtDNA genome of the Zhengyang yellow chicken.

Blood samples were collected from a Zhengyang yellow chicken farm in Zhumadian city, Henan Province, China. Genomic DNA was extracted from whole blood by standard phenol/chloroform methods. In addition, PCR for mtDNA fragments, library construction and next-generation sequencing, and *de novo* assembly were conducted as per previous publication (Chen et al., 2016). We followed caveats for quality control in mtDNA genome study in domestic animals (Shi et al., 2014). We scored the variants relative to the GenBank reference sequence under Accession No. AP003321 (Nishibori et al., 2005), and manually checked the bam file exported by Torrent Suite 5.0.2 to confirm the scored variants using Integrative Genomics Viewer (Thorvaldsdóttir et al., 2013).

The complete mitochondrial genome of the Zhengyang yellow chicken was 16 785 bp in length (GenBank Accession No. KX987152), with a base composition of 30.3% for A, 23.7% for T, 32.5% for C, and 13.5% for G, showing a high A+T content of 54.0%. Furthermore, the genome contained a typical structure, including a non-coding control region (D-loop), two ribosomal RNA genes, 13 protein-coding genes, and 22 tRNA genes. The arrangement of all genes was identical to that of *Gallus* *gallus* mtDNA (e.g., Huang et al., 2016; Liu et al., 2016). All proteins started with ATG, except for *COX1* (GTG). In addition, apart from eight tRNA genes (*tRNA^Gln^*, *tRNA^Ala^*, *tRNA^Asn^*, *tRNA^Cys^*, *tRNA^Tyr^*, *tRNA^Ser^*, *tRNA^Pro^*, and *tRNA^Glu^*) and one protein-coding gene (*ND6*), all other mitogenome genes were encoded on the H strand. Different genes shared different stop codons; for example, *ND1*, *COX2*, *ATPase8*, *ATPase6*, *ND3*, *ND4L*, *ND5*, *Cytb*, and *ND6* used TAA as a stop codon, *ND2* used TAG, *COX1* used AGG, and *COX3* and *ND4* used an incomplete stop codon "T– –".

**Figure 1:**
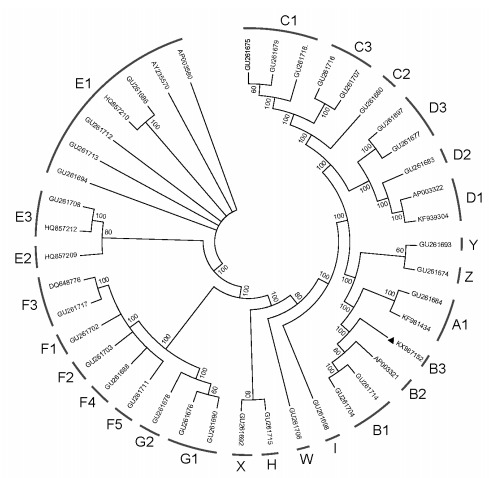
Phylogenetic tree based on mitochondrial genome analyses of 42 chicken samples using maximum parsimony

Phylogenetic analysis was performed using complete mtDNA sequences containing major haplogroups and sub-haplogroups, as defined by Miao et al. (2013) and Peng et al. (2015). The aligned sequences were analyzed by maximum parsimony using MEGA 5.0 with 1 000 bootstrap replicates (Tamura et al., 2011). Our results showed that the Zhengyang yellow chicken sequence was clustered with sequences belonging to haplogroup B (Figure 1). This newly generated sequence characterized a novel sub-haplogroup B3 within haplogroup B (Miao et al., 2013; Peng et al., 2015) (Supplementary Figure S1). This sub-haplogroup B3 was determined by an additional coding region variation at site 16 359. After searching the published chicken mtDNA datasets, we found seven chicken mtDNAs containing this variation, but they did not belong to B3 (data not shown).

Haplogroup B is common in chicken mtDNA datasets (Liu et al., 2006; Miao et al., 2013), but no geographic distribution information for sub-haplogroup B3, which was defined by both D-loop variants and coding region variations, currently exists. Identification of more B3 mtDNAs (by genotyping the variation at site 16 359 in those haplogroup B samples defined by the D-loop mutation motif) will provide additional information regarding the geographic origin and dispersal of this lineage in domestic chicken.

## ACKNOWLEDGEMENTS

The authors thank the Bureau of Animal Husbandry of Zhengyang County for their assistance in sampling.
